# Enzootic Rabies Elimination from Dogs and Reemergence in Wild Terrestrial Carnivores, United States

**DOI:** 10.3201/eid1412.080876

**Published:** 2008-12

**Authors:** Andrés Velasco-Villa, Serena A. Reeder, Lillian A. Orciari, Pamela A. Yager, Richard Franka, Jesse D. Blanton, Letha Zuckero, Patrick Hunt, Ernest H. Oertli, Laura E. Robinson, Charles E. Rupprecht

**Affiliations:** Centers for Disease Control and Prevention, Atlanta, Georgia, USA (A. Velasco-Villa, S.A. Reeder, L.A. Orciari, P.A. Yager, R. Franka, J.D. Blanton, C.E. Rupprecht); Texas Department of State Health Services, Austin, Texas, USA (L. Zuckero, P. Hunt, E.H. Oertli, L.E. Robinson)

**Keywords:** Rabies elimination, rabies re-emergence, molecular epidemiology, oral vaccination, rabies in wildlife, research

## Abstract

To provide molecular and virologic evidence that domestic dog rabies is no longer enzootic to the United States and to identify putative relatives of dog-related rabies viruses (RVs) circulating in other carnivores, we studied RVs associated with recent and historic dog rabies enzootics worldwide. Molecular, phylogenetic, and epizootiologic evidence shows that domestic dog rabies is no longer enzootic to the United States. Nonetheless, our data suggest that independent rabies enzootics are now established in wild terrestrial carnivores (skunks in California and north-central United States, gray foxes in Texas and Arizona, and mongooses in Puerto Rico), as a consequence of different spillover events from long-term rabies enzootics associated with dogs. These preliminary results highlight the key role of dog RVs and human–dog demographics as operative factors for host shifts and disease reemergence into other important carnivore populations and highlight the need for the elimination of dog-related RVs worldwide.

## Abstract 2

Independent enzootics in wild terrestrial carnivores resulted from spillover events from long-term enzootics associated with dogs.

Rabies virus (RV) is the prototype member of the genus *Lyssavirus*, which causes acute encephalomyelitis in mammals, including humans, throughout the world. The disease is independently maintained by several species of mammals within the orders Carnivora and Chiroptera (*1*). Intraspecies RV transmission maintains the disease within geographically discrete (*2*,*3*) areas by distinctive virus variants and lineages, which can be identified either by panels of monoclonal antibodies or by genetic analysis (*4*,*5*). Interspecies transmission of rabies often results in ecological bottlenecks and epidemiologic dead ends (*1*,*4*,*6*). However, during rabies epizootics or long-term enzootics, novel rabies reservoirs may emerge and produce outbreaks involving closely related RV variants (*7*,*8*).

Dog rabies has been recognized since the first human civilizations (*9*). However, the possible origins of disease in this species likely pre-date its direct associations with humans. Over time, RVs have had the opportunity to evolve within the context of the human–dog bond and to generate a suite of genetically distinguishable and geographically circumscribed variants and lineages (*10*–*12*). Dog RVs cause >55,000 human deaths annually, mostly in Asia and Africa (*13*). In the Americas, despite a 90% reduction of cases during past decade, the domestic dog still poses the greatest public health hazard with regard to rabies (*14*).

During the first half of the 20th century, canine rabies was enzootic throughout the United States (*15*). By the 1970s, the primary dog RV variant had been eliminated by extensive mass vaccination programs and effective control of stray animal populations (*1*,*15*). During the late 1980s, the domestic dog–coyote RV variant became enzootic in the coyote (*Canis latrans*) population along the United States–Mexico border (*16*). In 1994, this variant expanded by translocation into Alabama and Florida, causing a local outbreak in domestic dogs (*17*). In 1988, a second canine RV variant, the Texas gray fox variant, was found associated with enzootic rabies in gray foxes (*Urocyon cinereoargenteus*) in west-central Texas (*16*,*17*). Aggressive oral vaccination programs in the region eliminated the domestic dog–coyote RV variant by 2004, while circulation of the Texas gray fox variant was substantially constrained (*18*). Thus, the United States became the most recent country to eliminate enzootic dog rabies (*19*).

To provide molecular and virologic evidence that domestic dog rabies is no longer enzootic to the United States and to identify putative relatives of dog-related RVs circulating in other carnivores, we studied RVs associated with recent and historic dog rabies enzootics worldwide. We compared historical surrogates that circulated in the United States with RV isolates suspected of being dog related, collected over the past 15 years.

## Materials and Methods

### Viruses

A total of 228 samples were sequenced, generating both full and partial RV nucleoprotein gene data. The 152 samples from the United States were obtained from 136 dogs and coyotes from Texas during 1991–2007; 1 dog, 1 bat, and 1 skunk from New Mexico, 2006–2007; 1 gray fox from Arizona, 1986; 3 dogs from Florida, 1994; 1 dog from Alabama, 1994; 2 mongooses and 1 dog from Puerto Rico, 2004–2006; 1 dog from New York, 1949; 1 person from California, 1954; 1 coyote from San Diego, California, 1990 (*20*); 1 dog from California, 2007; and 1 dog from Alaska, 2007. Most samples were from Texas, where canine-associated RVs have been found, although believed to be independent of dog rabies, after enzootic dog rabies was eliminated from the United States during the 1970s (*1*,*15*). The 76 samples from other countries came from 58 dogs from Mexico, representing all dog-related lineages described to date during 1961–2003 (*20*,*21*); 2 gray foxes from Sonora and Chihuahua, 1994–2002 (*21*); 3 skunks from Baja California Sur, 1998–2007 (*20*,*21*); 2 skunks from Durango and Sonora, 2001–2003 (*20*,*21*); 1 dog from Honduras, 2001; 1 person from El Salvador, 2002; and 9 vampire bats from Mexico, 2002–2004. To entail a global perspective, we compared these sequences with historical and recent sequences associated with major dog and terrestrial wildlife rabies foci in the United States and elsewhere (*11*,*12*,*20*–*24*). Vaccine strains with known American origin were included as surrogates of enzootic dog RVs in the United States. They were represented by Flury strains low egg passage and high egg passage, originally isolated from a person in Georgia in 1939 (*25*) and by Street-Alabama-Dufferin B19 and Evelyn Rokitniki Abelseth derivatives, obtained from a rabid dog in Alabama in 1935 (*26*). Surrogates of enzootic dog RVs from the Old World were also incorporated in the global analysis. Pasteur’s derivatives, originally obtained from a rabid cow in Paris, France, in 1882, such as Pasteur virus, Pitman-Moore, an avirulent mutant derived from challenge virus standard AvO1, and challenge virus standard, were also considered in this regard (*27*).

### Sequence Analyses

We performed multiple alignments of the partial and entire nucleoprotein RV sequences by using ClustalW (www.ebi.ac.uk/clustalw/index.html). To verify the robustness of the phylogenetic inferences, we conducted the analysis by using phenetic and cladistic methods with MEGA 2.1 (*28*) and MrBayes (www.mrbayes.net) programs. Corrected nucleotide substitutions were calculated by using the Kimura 2-parameter and general time reversible model for neighbor joining and MrBayes, respectively. Confidence limits were estimated by a bootstrap algorithm applying 1,000 iterations (*29*). To contrast RV associated with terrestrial carnivore species, we also used consensus sequences associated with bat rabies in Mexico and the United States to construct the phylogenies. We included other lyssavirus species as outgroups: European bat lyssavirus 1, European bat lyssavirus 2, and Australian bat lyssavirus (*11*,*23*).

## Results

### Elimination of Enzootic Dog RVs from the United States

**Figure 1:**
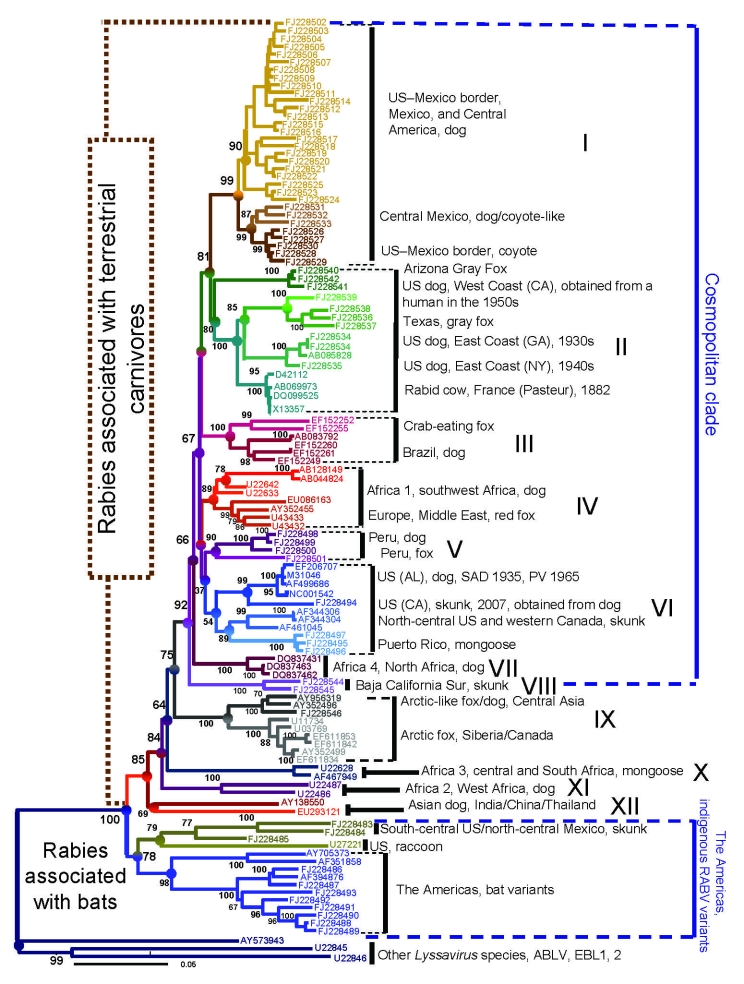
Neighbor-joining phyogenetic tree reconstructed by using entire nucleoprotein sequences that show the consensus topology observed with maximum-likehood and Bayes methods (www.mrbayes.net). The hierarchy encompassing phylogroups, clades, groups, lineages, and taxa of rabies viruses throughout the world is shown. SAD, Street-Alabama-Dufferin; RV, rabies virus; ABLV, Australian bat lyssavirus; EBL, European bat lyssavirus. Scale bar indicates number of nucleotide substitutions per site.

**Figure 2:**
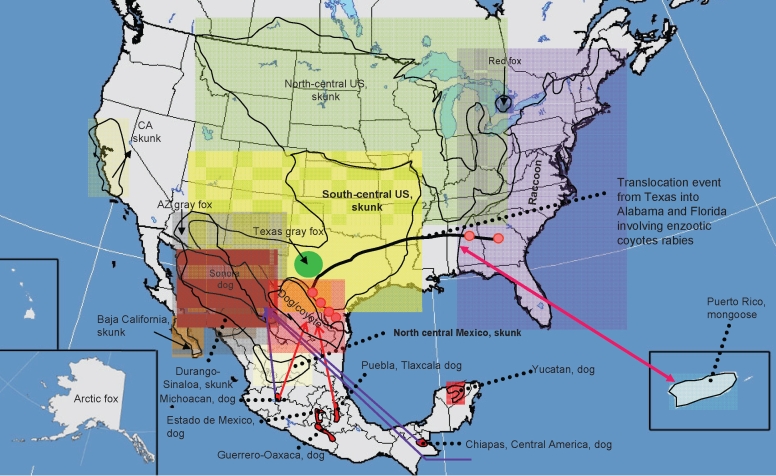
Current distribution of major rabies virus (RV) lineages associated with terrestrial carnivores and dogs in the United States and Mexico. Translocation movements proposed on the basis of the phylogenetic analysis (bidirectional arrows in colors) and confirmed translocations events on the basis of descriptive and epizootiologic investigations are shown. **Boldface** indicates RV lineages associated with rabies enzootics autochthonous for the New World (not associated with dogs).

The domestic dog–coyote RV variant lineage was found in a total of 122 samples from either dogs or coyotes. Most (118) of these were found in Texas during 1991–2004 (Figures 1, 2; Appendix Figure). The overall genetic identity among members of this lineage in the period analyzed was 98.9%. This lineage was monophyletic with an extinct enzootic dog rabies lineage distributed in central Mexico and west-central Mexico until the year 2000. An average identity value of 97.6% was noticed between these 2 lineages (Figure 2). A consensus amino acid change at position 134 (V replaced by I) within the nucleoprotein gene was observed in both lineages. However, a unique amino acid change at position 426 (S replaced by T) was characteristic for the domestic dog–coyote RVs. Similarly, the Sonora dog lineage, distributed along the western United States–Mexico border until 1994, was monophyletic with extant lineages circumscribed southward in west-central Mexico, southeastern Mexico (Chiapas), and Central America (Figures 1, 2). The overall genetic distance among samples within this lineage was 0.017 (98.3% identity). When compared with its closest relatives in southeastern Mexico and Central America, the identity value was 97.7% (Figures 1,2). An amino acid change at position 40 within the nucleoprotein gene (C replaced by S), common to dog-related rabies enzootics in Africa and Asia (Africa 2 and 3, arctic fox and Arctic-like, plus all dog-related lineages in Asia and India), was found in this lineage. Within the United States, the Sonora dog lineage was detected in 2 dogs, 2 coyotes, and 1 bobcat in counties bordering northeast Mexico in 1993, 1995, and 1996, respectively, and in 1 dog in San Diego, California, in 1990.

The domestic dog–coyote RV variant and Sonora dog lineage enzootics were expected to be related because of their geographic proximity (Figure 2). However, the phylogenetic data demonstrated that the Sonora dog lineage originated from independent dog rabies enzootics translocated from Central America, Honduras, Nicaragua, and Michoacán in west-central Mexico (Figures 1, 2).

The Texas gray fox RV variant was detected in Texas only, from 8 dogs and 3 coyotes during 1991–1995, and in 20 coyotes in the western part of Texas during 2007 (Appendix Figure). Members of the Texas gray fox variant or lineage presented a consensus amino acid change within the nucleoprotein gene at position 247 (K replaced by R) and an overall identity value of 99.1%. In 1986, a rabid gray fox from Arizona was found with the Arizona gray fox RV variant. This variant was also found circulating in northwestern Mexico (Figures 1,2); 3 consensus amino acid changes within the nucleoprotein gene at positions 9 (K replaced by R), 13 (Q replaced by H), and 421 (I replaced by V) characterized Arizona gray fox RV variant samples among all other extinct and extant enzootic canine-related variants and lineages circulating throughout the world. The average identity value for members of the Arizona gray fox RV variant lineage was 98.8%.

The Texas gray fox RV variants are enzootic to Texas, and the Arizona gray fox RV variants are enzootic to Arizona and northwestern Mexico, respectively; average identity value between variants is 92.9% (Figure 2). Distinctive consensus amino acid and nucleotide changes at the nucleoprotein gene have become fixed and have remained stable over time in these RVs circulating in gray fox populations in the southern United States. All lineages were monophyletic with historic RV (Flury high egg passage and low egg passage, CA human 1954, and NY dog 1949) obtained from virus repositories or vaccine strains that were originally recovered from canine rabies cases that occurred in the United States before dog rabies elimination (Figure 1). The topology in group II (Figure 1) details the circulation of at least 2 canine rabies lineages representing different dog rabies enzootics (before dog rabies elimination in the United States): one closely related to the Texas gray fox variant circumscribed in California (CA human 1954) and the other widely circulating over the East Coast, related to the Flury and NY dog sequences. This group also encompassed surrogates of enzootic dog rabies in Europe, which indicated an epidemiologic link between dog rabies epizootics on both continents (Figure 1).

The Puerto Rico mongoose RV variant was found in 2 mongooses and 1 dog. This lineage presented a homogeneous identity value of 99.1% and had common amino acid changes with Africa 1b and Africa 3 at position 254 (R replaced by K) and with the Arctic-like and Street-Alabama-Dufferin B19 RV at position 135 (S replaced by P), consistent with an Old World origin of these viruses. A characteristic amino acid change for this lineage was found at position 181 (I replaced by V). This variant was monophyletic with 2 North American lineages: the north-central skunk and the west Canada skunk RV. These skunk lineages, and the Puerto Rico mongoose lineage, were also monophyletic with Street-Alabama-Dufferin B19 derivatives and with a California skunk lineage, integrating group VI, which presented a low bootstrap value (Figure 1). Nonetheless, group VI was consistent when different methods for phylogenetic reconstruction were used (maximum likelihood and Bayes data are available on request). Skunk RVs in the north-central United States, southwestern Canada, and California were monophyletic with Street-Alabama-Dufferin B19 and Pasteur virus strains, both surrogates for enzootic dog rabies before its elimination in the United States and Europe, respectively. These results clearly illustrate the association of enzootic skunk rabies with historic dog rabies enzootics (Figure 1).

To underscore the importance of transcontinental translocation of dog-related rabies into the United States and also to illustrate that such phenomena still occur in the 21st century, during 2007 we studied a rabid dog imported from India into Washington state and then transported into Alaska. The infecting strain was determined to be the arctic fox–like variant, which is mainly enzootic in dog populations in northern India and central Asia (Figure 1).

Conversely, interspecies transmission of rabies into domestic dogs and wildlife was also observed in this study. Two dogs, 1 from Texas and the other from New Mexico, were typed as the *Tadarida brasiliensis* RV variant, whereas 1 coyote from Texas was identified with the south-central skunk RV variant. These findings illustrate that the interspecies transmission of rabies is a process that also may occur with regularity.

### Canine and Wildlife Rabies in the United States within a Historical and Global Context

To better understand dog rabies epizootiology in the United States, we also conducted a global analysis. Samples were divided into 2 main phylogroups, 1 associated with terrestrial carnivores and the other associated with bat rabies in the Americas. The terrestrial phylogroup was integrated by the cosmopolitan clade (CosC) that comprised at least 8 monophyletic groups (I to VIII, Figure 1). CosC encompassed 2–4 lineages representing independent enzootics associated with not only different species of Canidae (domestic dogs, jackals, wolves, gray foxes, red foxes, arctic foxes, raccoon dogs, Corsac foxes, crab-eating foxes, coyotes) but also with other species of terrestrial carnivores (mainly skunks, such as the Baja California skunk in Mexico and California skunks in the United States). Each of these groups consistently presented at least 1 lineage associated with rabies enzootics in domestic dogs (either extinct or extant). The remaining lineages were associated with rabies enzootics in terrestrial carnivores. The exceptions were groups VII and VIII, associated with either domestic dogs (Africa 4) or the Baja California spotted skunk *Spilogale putorious* (Figure 1). These phylogenetic patterns were consistent throughout Europe, Africa, and the Americas, where enzootic dog rabies was introduced (Figure 1). In addition, several other independent dog-related RV lineages were geographically circumscribed in different countries of the Americas, such as Argentina, Colombia, Bolivia, Peru, Ecuador, Nicaragua, and the Dominican Republic, and belong to the CosC as well (only partial nucleoprotein sequences available; tree available from the authors on request).

The average genetic distance among all members within CosC was 0.071%, equivalent to 93% average identity. The highest genetic distance observed between groups pertaining to this clade was 0.104 (89.6% average identity) and occurred between group III and group VIII (Figure 1). Meanwhile, the lowest genetic distance observed was between group I and group II 0.073 (92.7% average identity), followed by the same genetic distance of 0.074 (92.6%) between groups IV–V, IV–VI, and V–VI (Figure 1). The terrestrial phylogroup also encompassed independent groups and lineages associated with enzootic dog rabies in Asia (this group was the most divergent with an average genetic distance of 0.126, 87.4%, encompassing lineages from China, Thailand, Vietnam, and India), Africa (Africa 2, group IX with an average genetic distance of 0.079, 92.1%), the arctic fox and the arctic fox–like lineages (Arctic/Arctic-like group IX, with an average genetic distance of 0.044, 95.6%), and the African mongoose lineage (Africa 3, with an average genetic distance of 0.021, 97.9%).

Conversely, the bat phylogroup comprised RV lineages and variants associated with independent rabies enzootics maintained by different species of bats and 2 independent enzootics established in raccoons (raccoon variant) and skunks (south-central US skunk and Mexican north-central skunk), which may represent autochthonous RV lineages in the New World (Figures 1, 2). These lineages have an average genetic distance of 0.155 (84.5%) to 0.158 (84.7%) when compared with RV lineages associated with the terrestrial carnivore clade.

## Discussion

On the basis of phylogenetic analysis in conjunction with historic and recent epizootiologic data on rabies, we found no evidence of enzootic dog rabies in the United States for the past 13 years (*1*,*15*,*16*,*18*,*19*,*30*). Our findings suggest that independent rabies enzootics are now established in wild terrestrial carnivores (skunks in California and the north-central United States, gray foxes in Texas and Arizona, and mongooses in Puerto Rico), as a consequence of different spillover events from the long-term rabies enzootics associated with dogs (*31*).

The concept of dog-related lineages spilling into wildlife species with further host shifts was consistent for all phylogentic groups shown as part of CosC (I to VI) (*8*,*10*,*21*–*24*,*31*–*34*). This tendency was also consistent for other groups outside the CosC, such as group IX (*35*,*36*). Dog rabies epizootics in the Americas seem to be characterized by the circulation of multiple geographic or temporal lineages that have either become extinct or merged over time (*2*,*3*,*8*,*10*,*21*,*22*,*32*–*34*). Such a tendency was clearly observed in groups I, II, and VI and was implicit in groups III, IV, V, and VII (*2*,*3*,*5*,*8*,*10*,*12*,*21*–*23*,*31*–*33*,*35*,*36*). After European colonization in the 15th century, multiple dog-related RV lineages have been introduced and evolved over time in the United States and elsewhere in the Americas (*5*,*8*,*10*–*12*).

The historical background in Europe and the Middle East supports the suggestion that enzootic red fox rabies was derived from a dog rabies epizootic in Europe (*5*,*8*,*10*–*12*). However, none of the surrogates representing European enzootic dog rabies early in the 19th century (Pasteur virus, Pitman-Moore, AV01, or challenge virus standard) segregated with enzootic RVs circulating in European wild terrestrial carnivores. These observations suggest that host-switching events may have occurred from other likely extinct dog RV lineages or, alternatively, from extinct lineages contemporary to Pasteur virus yet pertaining to independent dog rabies enzootics (*8*,*10*–*12*).

There is no cultural, historical, or epidemiologic evidence of enzootic dog rabies in the New World before European colonization (*8*,*10*). One of the oldest rabies enzootics associated with wild canines in North America occurred in arctic and red foxes (*37*,*38*). This fox rabies enzootic was found to be associated with the expansion of arctic fox rabies from northern Asia (*35*). We did not find any direct phylogenetic linkage between CosC and arctic fox rabies, as exists between arctic fox–like rabies epizootics in Asia and arctic fox rabies prevailing in the circumpolar Arctic region (*36*).

The global origin of enzootic rabies in dogs and in other terrestrial carnivores remains unknown but likely pre-dates the origin of dogs 15,000–100,000 years ago (*39*). A higher mitochondrial DNA haplotype diversity found in dog breeds from eastern Asia correlates with a higher genetic diversity of dog-related RV lineages in the same region (*8*,*10*,*35*,*36*,*39*). The latter observations raise questions about the possible coevolution of wild canines and RVs, the role of dog domestication in the dissemination of rabies, and the emergence of rabies enzootics in other wild terrestrial carnivores elsewhere. Nonetheless, independent rabies enzootics that persist in raccoons in the eastern United States and among skunks in the south-central United States and north-central Mexico seem to have their origin in RVs associated with indigenous bats species (*8*,*31*).

Global migration, social factors, and trading activities continue to promote and enhance long-distance movements of rabies-infected animals, increasing the potential risk of reintroducing the disease to regions where the problem has been eliminated. This trend poses new challenges for the regulation of animal movements to attain sustainable elimination.

Clearly, canine rabies elimination is needed on a global level. Vaccination of dogs should be maintained until all dog-related lineages and biotypes currently circulating in wildlife have been eliminated. The canine origin of these viruses makes them prone to return to dogs, where the disease may easily become enzootic again without proper attention related to laboratory-based surveillance, prevention, and control.

## Supplementary Material

Appendix Figure Neighbor-joining phylogenetic tree reconstructed by using partial nucleoprotein sequences that depict all samples from the southern United States, where canine enzootics have been eliminated within the 21st century. The imported Alaska dog from 2007 is also shown within the Arctic-like and arctic fox group in gray. RVs, rabies viruses.
