# Supplementary figures and images for: Enzootic Rabies Elimination from Dogs and Reemergence in Wild Terrestrial Carnivores, United States
Source: Emerg Infect Dis. 2008 Dec;14(12):1849–54. doi: 10.3201/eid1412.080876 (PMC2634643; doi:10.3201/eid1412.080876)

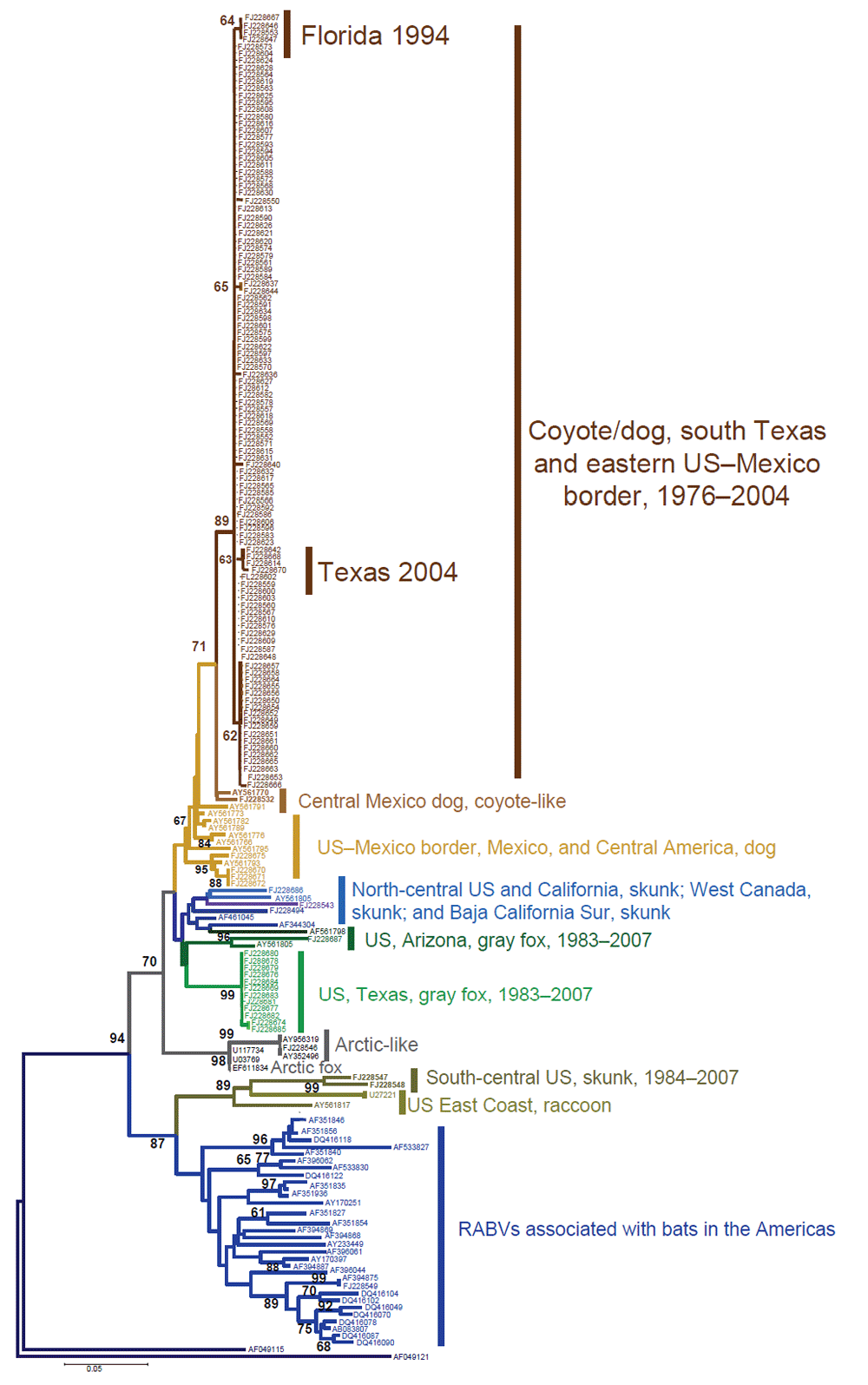

Supplement: Appendix Figure — Neighbor-joining phylogenetic tree reconstructed by using partial nucleoprotein sequences that depict all samples from the southern United States, where canine enzootics have been eliminated within the 21st century. The imported Alaska dog from 2007 is also shown within the Arctic-like and arctic fox group in gray. RVs, rabies viruses. [file 08-0876_app-s1.gif]
